# Supplementary material for: Causal Relationship between Aging and Anorexia Nervosa: A White-Matter-Microstructure-Mediated Mendelian Randomization Analysis
Source: Biomedicines. 2024 Aug 16;12(8):1874. doi: 10.3390/biomedicines12081874 (PMC11351342; doi:10.3390/biomedicines12081874)
Supplement: Supplementary file 1 [file biomedicines-12-01874-s001.zip › Supplementary Figure/Figure S4/Singe SNP plot of MR analysis for TL to ubm-b-1755.pdf]

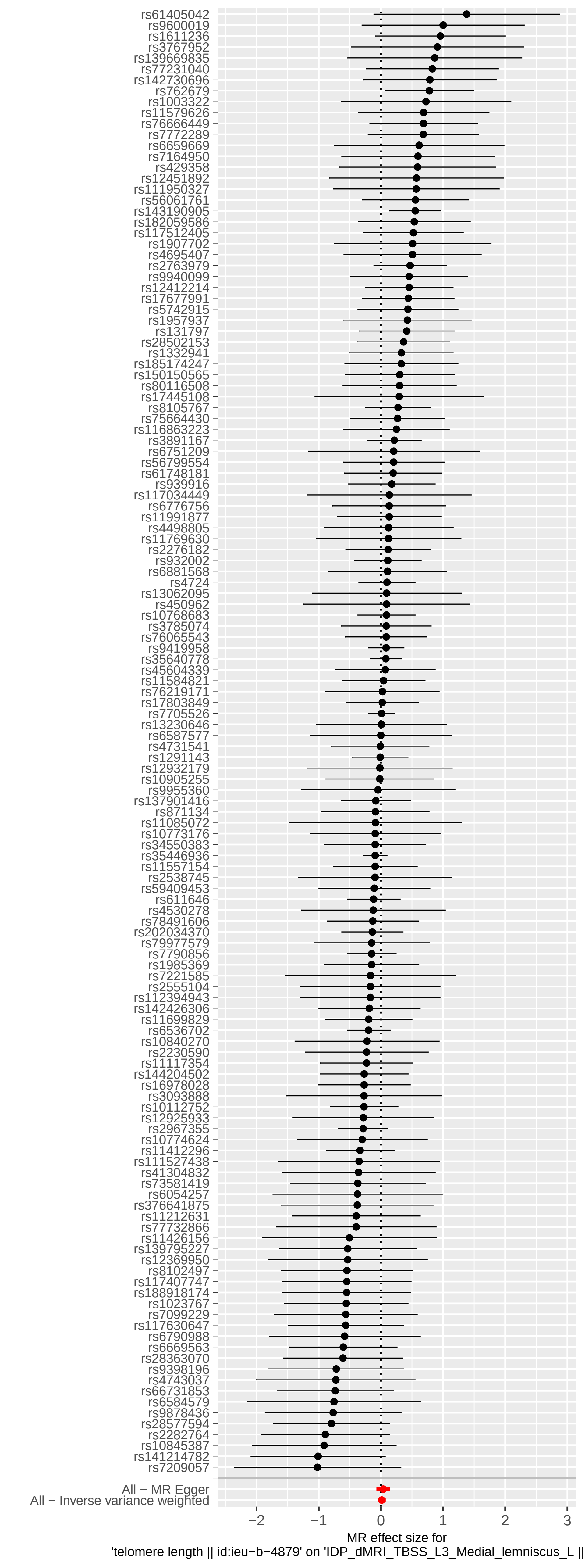
rs61405042
rs9600019
rs1611236
rs3767952
rs139669835
rs77231040
rs142730696
rs762679
rs1003322
rs11579626
rs76666449
rs7772289
rs6659669
rs7164950
rs429358
rs12451892
rs111950327
rs56061761
rs143190905
rs182059586
rs117512405
rs1907702
rs4695407
rs2763979
rs9940099
rs12412214
rs17677991
rs5742915
rs1957937
rs131797
rs28502153
rs1332941
rs185174247
rs150150565
rs80116508
rs17445108
rs8105767
rs75664430
rs116863223
rs3891167
rs6751209
rs56799554
rs61748181
rs939916
rs117034449
rs6776756
rs11991877
rs4498805
rs11769630
rs2276182
rs932002
rs6881568
rs4724
rs13062095
rs450962
rs10768683
rs3785074
rs76065543
rs9419958
rs35640778
rs45604339
rs11584821
rs76219171
rs17803849
rs7705526
rs13230646
rs6587577
rs4731541
rs1291143
rs12932179
rs10905255
rs9955360
rs137901416
rs871134
rs11085072
rs10773176
rs34550383
rs35446936
rs11557154
rs2538745
rs59409453
rs611646
rs4530278
rs78491606
rs202034370
rs79977579
rs7790856
rs1985369
rs7221585
rs2555104
rs112394943
rs142426306
rs11699829
rs6536702
rs10840270
rs2230590
rs11117354
rs144204502
rs16978028
rs3093888
rs10112752
rs12925933
rs2967355
rs10774624
rs11412296
rs111527438
rs41304832
rs73581419
rs6054257
rs376641875
rs11212631
rs77732866
rs11426156
rs139795227
rs12369950
rs8102497
rs117407747
rs188918174
rs1023767
rs7099229
rs117630647
rs6790988
rs6669563
rs28363070
rs9398196
rs4743037
rs66731853
rs6584579
rs9878436
rs28577594
rs2282764
rs10845387
rs141214782
rs7209057
All − MR Egger
All − Inverse variance weighted
−2
−1
0
1
2
3
MR effect size for
'telomere length || id:ieu−b−4879' on 'IDP_dMRI_TBSS_L3_Medial_lemniscus_L ||
